# Supplementary material for: Mortality rate and associated factors among patients co-infected with drug resistant tuberculosis/HIV at Mulago National Referral Hospital, Uganda, a retrospective cohort study
Source: PLOS Glob Public Health. 2023 Jul 6;3(7):e0001020. doi: 10.1371/journal.pgph.0001020 (PMC10325059; doi:10.1371/journal.pgph.0001020)
Supplement: Attachment — Submitted filename: Joan Reviewers comment final.docx [file pgph.0001020.s001.docx]

Dear Sir,

I would like to thank you and the reviewers for the comments. My submission was delayed because we got an outbreak of Ebola Virus Disease in Uganda (9th September 2022) and i was chosen to be part of the senior epidemiologists to be on the forefront, as such it took most of my time and it's still taking the time . I am delighted to address the reviewers’ comments and hopefully get my paper published. The comments were addressed as follows.

| Comment | Action |
| --- | --- |
| Considering that this is a cohort study examining the mortality rate, I am curious as to why the authors chose modified poisson methods to determine the rates rather than calculating incidence rates (per person years). I would advise a survival analysis and Cox regression models, unless there is a compelling reason not to. | Because the study was looking at mortality rate, Modified Poisson regression was chosen as the most suitable method of analysis because it describes counts per unit time (rate) rather than survival analysis and cox regression that looks at time as the response variable that has elapsed between some origin and an event of interest. |
| The descriptions of study participants and inclusion criteria are ambiguous. Does the study solely include DR-TB/HIV patients taking second line TB drugs? OR were participants just newly diagnosed with DR-TB? | The study included patients that were confirmed to have all the two infections i.e., DR-TB and HIV with a recorded outcome (Died or Alive) and had attended the Mulago Tuberculosis clinic between January 2014 to December 2019  The studied included all DR-TB/HIV co-infected patients that were newly diagnosed or were on second line TB drugs. |
| It would be very helpful and in accordance with the STROBE standard to create a study flow diagram. | Study flow diagram is included in this manuscript |
| If possible, I would like the authors to provide the following significant DR-TB and HIV infection characteristics: a. Drug resistant profiles, including mono, poly, MDR, XDR, Rifampicin or otherwise. b. Type of TB (pulmonary, extra-pulmonary, or both) c. Location of TB d. Diagnosis order (TB first, HIV first, or diagnosed at the same time), time between HIV and TB infection e. TB medications specifically (moxifloxacin, levofloxacin, bedaquiline, or linezolid: they are associated with significantly decreased odds of death.) | Data on TB location, Type of TB, Location has been included in the analysis. HIV status was documented in the records at baseline but it is unclear which of the two diagnoses came first for the patient and all patients had rifampicin resistance at baseline (include this in the results).  Analyses on TB medications like (moxifloxacin, levofloxacin, bedaquiline, or linezolid have been included |
| A nice meta-analysis and some sub-analyses on MDR-TB and HIV mortality were provided by Bisson et al. in 2020 (<https://doi.org/10.1016/S0140-6736(20)31316-7>), which may guide further revision of the analyses in this manuscript. | Paper reviewed and it guided me on further refining and revision of my manuscript |
| MUAC cut-off point appears to be poorly correlated with BMI categories, suggesting a severe illness or acute malnutrition. I would suggest lowering the cut-off point to roughly 18.5, which some say is appropriate for all settings, to accurately reflect this position. (Global MUAC cut-offs for adults: Technical Consultation: <http://pdf.usaid.gov/pdf_docs/PA00T7K9.pdf>) | MUAC cut offs lowered to 18.5 cm and data reanalysed as per the Global MUAC cut-offs for adults. |
| It doesn't seem necessary to include Figure 2. Include it as text in the Results section. | Figure two deleted and summarised as text in the results section |
| Table 2 and 3 should be presented in one table. | Table 2 and 3 were merged and presented as one in the manuscript |
| Were there any changes in the mortality rate during the years of the study? This information could help show any trend. | A graph showing the trends of death by year has been incorporated. |
| Other variables can impact the mortality outcome, for example, CD4 count, viral load, localization of TB (pulmonary or extrapulmonary TB), etc. Could you let me know if you collected this information? If not, Why not? | Information on some variables such as viral load, TB localisation (pulmonary or extrapulmonary TB), ART regimen were collected and they have been included in the analysis |
| Is the median time to mortality reported? E.g., time since diagnosis or time since treatment initiation | The median time to mortality is time since treatment initiation |
| Please explain why you decided to divide the age into two categories. | The age was based upon the mean of the population thus it was categorized around the mean |
| How many patients were included in the final model? Please clarify. | 375 patients were included in the final model |
| The authors mention they checked interaction among the variables; please specify what interaction you checked and what are the results. | Interaction terms for all the variables were created and two models were created. The Full model with variables and interaction terms and a reduced model that had only variables. A chuck test was run by subtracting -2Log Likelihood of full model from -2Log Likelihood of the reduced model considering the degrees of freedom. There was no interaction |
| You used the BMI and MUAC in the same model. Are those anthropometric measures related? Did you evaluate the collinearity? | BMI and MAUC may have a positive correlation, however, in this study they were not collinear. A linear regression model of the two variables was run and the variance inflation factors of both BMI and MUAC were computed. Both VIFs were 1, which signified that there is no correlation between BMI and MUAC |
| You mention the study was carried out in the Mulago Hospital tuberculosis unit. Having access to the patient files, why did you not check for other comorbidities that could be associated with mortality, like diabetes? If you did, I think you should mention it and include this information in the paper. If not, why not? | Information pertaining to other underlying comorbidities was not included in the patient TB register as such it was included as a limitation in this study.  Additionally, the prevalence of some of the comorbidities like DM is very low. |
| The authors review the mortality rates for DR-TB/HIV reported by other publications showing lower mortality rates in coinfected patients. Given this, I suggest that the authors discuss in depth the implications that these high rates represent for the country and issue some recommendations. | Implications of a high mortality rate for the Uganda settings included and recommendations appended. |
| Were there any changes in the TB treatment administration during the study? In addition, the authors mention, "This is because, by the time this study was undertaken, antiretroviral treatment (ART) was rolled out countrywide and was available in Uganda". Was this change during the first year of the study? | Yes there were changes in TB drug administration during the study The DRTB treatment guidelines have evolved over time.  The programmatic management of DR-TB in Uganda began in 2012  although a local guideline was in place in 2011. The guidelines  recommended a standardised regimen that consisted of a 6-months’  intensive phase with kanamycin (Km) (or capreomycin), levofloxacin  (Lfx), ethionamide (Eto), cycloserine (Cs), and pyrazinamide (Z)  followed by a continuation phase of 18 months without the aminoglyco-side. Individualised regimens were recommended when informed by drug susceptibility testing and tolerance. The alternative agents were ethambutol (E), amikacin (Am) and p-amino salicylic acid (PAS). In 2016, the revised guidelines recommended an intensive phase of 6 months of Km + Lfx + Eto + Cs + Z or 4 months after culture conversion, whichever was longer. A continuation phase without the aminoglyco-side for a duration of 14 months or at least 20-months post-culture conversion (whichever was longer), was recommended. Moxifloxacin (Mfx) was recommended as an alternative to Lfx, while Cfz and Lz were recommended as possible options to Eto. Bedaquiline and delamanid, newly discovered drugs for DR-TB treatment, were initially available on a compassionate basis until they were increasingly available in the country by 2018. A modification to the standard regimen was recommended for the treatment of XDR-TB and pre-XDR – TB (MDR – TB with additional resistance to either a fluroquinolone or second line injectable  aminoglycoside) but the decision was to be made by the national DR –  TB panel on a case-to-case basis. The national DR-TB panel also  provides guidance on other difficult-to-treat patients. In 2017, an annex to the 2016 guidelines introduced the short-term regimen (STR) which consisted of 4–6 months of Km + Mfx + Cfz + Z + E + H high dose + Eto and 5 months of Mfx + Cfz + Z + E for patients with confirmed sensitivity to fluoroquinolones and an injectable aminoglycoside. Treatment was administered by health worker supervised directly observed therapy (DOT) using both facility and community-based models.  ART was rolled out in 2004 in Uganda so by the first year of the study it was rolled out. |
| Please check the spelling and spaces used in the document, including the tables | Spellings and spaces checked in the document and the tables |
| Could you update the references? For example, reference #1 is the WHO 2018 TB report; there is a new version (2021). | Reference updated |
| Please, check the results section regarding the date that the patients were admitted. There is a mistake there. | Mistake rectified. |
| Please, explain what MUAC means the first time it appears. | MUAC mid-upper arm circumference and has been updated and what it means where it appears first. |
| Review the structure of the references; some of them are incomplete | References reviewed |
| There are two references in the results (Mortality rate). What do those mean? | Because median is reported with the interquartile range, I had reported the median time to mortality with its IQR, but I removed it |
